# Minimal Increase Network Coding for Dynamic Networks

**DOI:** 10.1371/journal.pone.0148725

**Published:** 2016-02-11

**Authors:** Guoyin Zhang, Xu Fan, Yanxia Wu

**Affiliations:** College of Computer Science and Technology, Harbin Engineering University, Harbin, Heilongjiang, P. R. China; Semmelweis University, HUNGARY

## Abstract

Because of the mobility, computing power and changeable topology of dynamic networks, it is difficult for random linear network coding (RLNC) in static networks to satisfy the requirements of dynamic networks. To alleviate this problem, a minimal increase network coding (MINC) algorithm is proposed. By identifying the nonzero elements of an encoding vector, it selects blocks to be encoded on the basis of relationship between the nonzero elements that the controls changes in the degrees of the blocks; then, the encoding time is shortened in a dynamic network. The results of simulations show that, compared with existing encoding algorithms, the MINC algorithm provides reduced computational complexity of encoding and an increased probability of delivery.

## Introduction

Network coding, which was first proposed in the context of information theory, has been introduced into communication networks to improve transmission efficiency and to ease block propagation scheduling [1, 2]. It has been shown that network coding enables a wide variety of applications, including communications in ad-hoc networks and data collection in sensor networks, by exploring the broadcast essence of wireless networks [3, 4]. Since the block is the universal component of network coding, a node can transmit any encoded block to other nodes, and each block has a high probability of equal contribution to the nodes.

RLNC [5] has been proven to be a powerful tool for disseminating information over a network. According to the previous studies of RLNC [2, 5], encoding operations are performed by both the sources and the intermediate nodes. Whenever a node or a source needs to forward a block to another node, it simultaneously produces a linear combination of all of the blocks in storage.

Even though RLNC greatly facilitates network implementation by providing a decentralized solution, the relatively high complexity of encoding at sources and intermediate nodes remains a major obstacle to its widespread use. To reduce the complexity of the coding process, Chou et al. [6] proposed the concept of group network coding, in which blocks are encoded within the same group or segment, with each segment containing a prescribed number of blocks. This coding scheme, i.e. using all of the plain blocks in a given segment to generate encoded blocks, is very highly complex and requires a great deal of overhead for disk operation. The sparse form of RLNC (Paircoding), in which only two random plain blocks are used to generate new blocks, is proposed in [7]. Paircoding is regarded as at least as good as BitTorrent for file sharing, and the two have nearly the same computational complexity in terms of disk access. However, Paircoding cannot be fully exploited because of the low rate at which it generates new blocks. Cai et al. [8] analyzed the potential for improvement in the content made available and bandwidth used by two existing network coding schemes and proposed a sparse network coding scheme to accelerate the generation of new blocks. From the perspective of group network coding, each segment in this coding scheme contains two plain blocks. Niu and Li [9] quantitatively evaluated how network coding can be used to improve content availability, block diversity, and download performance in the presence of churning as the number of blocks varies in each coding segment. To reduce the complexity of decoding, Feizi et al. [10] noted the need for nodes in different transmission phases and designed a tunable sparse network coding algorithm in which the density of the network coded packets varies during a transmission. An encoding window [11] is an effective tool for controlling the computational complexity of an encoding operation that encodes only the data that have a nonzero component within the window. As Paircoding does, an encoding window reduces the computational complexity while limiting the generation speed of encoding a block.

We revisit the idea of using sparse coding to reduce the complexity of the encoding process via a fundamentally different approach in which a newly generated block is deployed to maintain a minimal difference from the existing blocks. This scheme is named MINC. The remainder of this paper is organized as follows: In Section 2, a brief introduction is offered to the basic mathematical description of RLNC. Section 3 will witness our proposal of the MINC scheme and the analysis of its probabilistic properties. Section 4 is spared for an experimental evaluation of the encoding speed and distribution efficiency of MINC. Finally, Section 5 concludes the whole paper with a summary of the research findings.

## Model and Preliminaries

In this section the principles will be delineated behind RLNC. Then, it will be demonstrated how random packets get recombined at the nodes of a randomly connected graph. The following part takes into account of a general multicast network with one source and several receivers. Then the network will be modeled as a directed graph, *G* = (*V*, *E*), where *V* is the set of nodes in the network and *E* is the set of edges in *G*, *E* is a subset of *V* × *V* which represent point-to-point channels.

The source node holds a message, *F*, i.e. a generated file, that must be distributed to multiple network nodes. In RLNC, the file is divided into *G* segments, each of which is further broken into *m* blocks in the source node. If segment *i* has an original block, $B^{i}={\left[\begin{array}{cccc} B_{1}^{i} & B_{2}^{i} & \cdots & B_{m}^{i} \end{array}\right]}^{T}$, then, an encoded block, *b*^*i*^, from segment *i* is a linear combination of ${\left[\begin{array}{cccc} B_{1}^{i} & B_{2}^{i} & \cdots & B_{m}^{i} \end{array}\right]}^{T}$ in the Galois field *GF*(*q*), i.e. $b^{i}=\sum_{j=1}^{k}c_{j}B_{j}^{i}$ 1 ≤ *j* ≤ *m*,*c*_*j*_ ∈ *GF*(*q*).

If a node has *l*(*l* ≤ *m*) encoded or un-encoded blocks of segment *i*, $b={\left[\begin{array}{cccc} b_{1}^{i} & b_{2}^{i} & \cdots & b_{l}^{i} \end{array}\right]}^{T}$, then, when serving another node, *p*, it randomly selects a set of coding coefficients from GF(*q*), namely, the coding vector denoted by *c* = [*c*_*1*_ *c*_*2*_ ⋯ *c*_*l*_], and then, encodes all of the blocks in segment *i* and produces one encoded block, $x^{i}=\sum_{j=1}^{l}c_{j}\cdot b_{j}^{i}=c\cdot b$. The encoding vector of the new block is denoted by **y**, where ***y*** = (*y*_*1*_ *y*_*2*_ ⋯ *y*_*n*_) = (*c*_*1*_ *c*_*2*_ ⋯ *c*_*n*_). We use the encoding vector to represent the corresponding block in the following sections.

In an encoded block, *x*, the coding coefficients used to encode original blocks are embedded in the header of the encoded block. As soon as a peer, *p*, successfully receives a total of *m* encoded blocks from segment *i*, $x={\left[\begin{array}{cccc} x_{1}^{i} & x_{2}^{i} & \cdots & x_{m}^{i} \end{array}\right]}^{T}$, that are linearly independent, it is able to decode segment *i* with the coding coefficients embedded in each of the *m* encoded blocks. To decode segment *i*, we must first compute the inverse of the coefficient matrix **A**_***i***_ by Gaussian elimination. To obtain the original *m* blocks, it is then necessary to multiply $A_{i}^{-1}$ and **x**, i.e. **B***^i^* = **A**^−1^×**x**.

## Minimal Increase Network Coding

As described in the section on the coding process, we know that RLNC adopts a high-density encoding scheme to ensure that the encoding vectors are linearly independent, which leads to excessive computational overhead during the encoding process. From the perspective of decoding, the relationship among the encoding vectors, not the coding density, determines whether a receiver can decode a source file.

**Fig 1:**
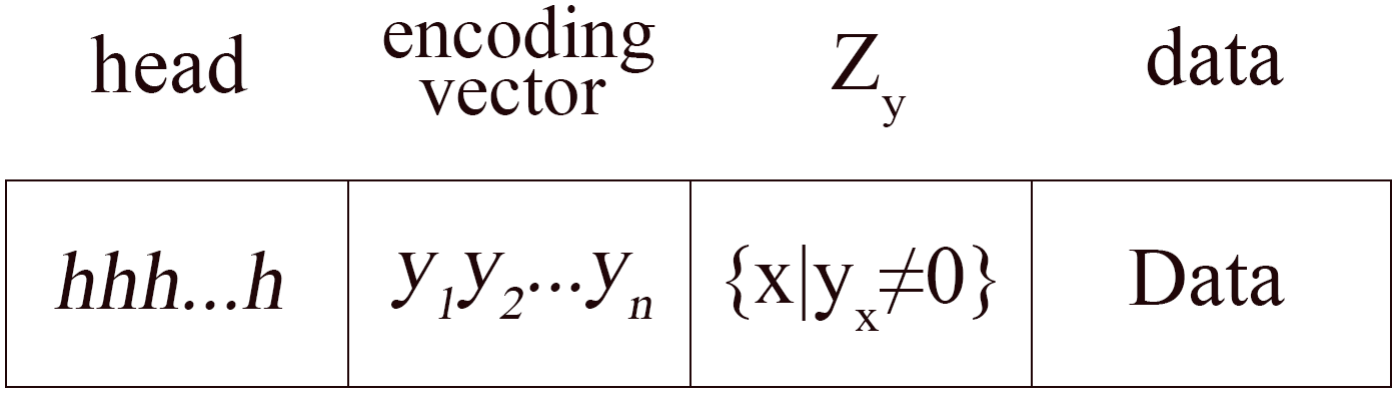
The MINC data packet format.

The nonzero elements of an encoding vector are denoted by the set *Z*_*y*_, i.e. Z_y_ = {*x*| *y*_*x*_ ≠ 0}. For any two encoding vectors **y** and **y**′, if Zy\Zy′≠∅, we say that **y** is not less than **y**′ and write **y ▷ y′**. Intuitively, **y ▷ y′** explains that **y** contains nonzero elements that differ from those of y′. The term “degree” is employed to represent the number of nonzero elements in an encoding vector; the degree of an encoding vector is represented by *d*(**y**). When **y** = 0, *d*(**y**) = 0. The average degree of the encoding vectors in node *v* is denoted by $\bar{d}$. To facilitate processing, we add *Z*_*y*_ to the area in the encoded packet; the packet format is shown in Fig 1.

A source node, *S*, randomly selects two plain blocks for each encoding operation, and the encoding coefficients are selected from *GF*(2^*q*^)\{0}. The intermediate nodes control the process of choosing which block to encode based on its degree and relationship to *Z*_*y*_. One difference between the MINC and the RLNC methods is that in the former, node *v* saves the encoded block as a benchmark after transmitting it. The encoding vector of a benchmark block is denoted by **y**^**0**^, and the degree of **y**^**0**^ is denoted by *d*_0_. Before encoding the next block, node *v* selects a block that satisfies the condition **y′ ▷ y**^**0**^ to be encoded. The encoding coefficients are selected from *GF*(2^*q*^)\{0} by the source node. Then, the benchmark block is updated with the help of the newly encoded block. When $d_{0}>\bar{d}$, node *v* selects a block that satisfies $d\leq \bar{d}$ as the new benchmark block. The proposed encoding algorithm is shown in Algorithm 1.

**Algorithm 1.** Minimal Increase Network Coding for Intermediate Nodes.

**Input: y**^**i**^ (1 ≤ *i* ≤ *k*), **y**^**0**^

**Output: y′**

 1: **if** (*d*(**y**^0^) = 0 or $d(y^{0})>\bar{d}$) **then**

 2:  **y**^**0**^**←y**^**i**^ of $d\left(y^{i}\right)\leq \bar{d}$

 3: **end if**

 4: **while y**^**i**^ **◁y**^**0**^ **do**

 5:  **i++**

 6: **end while**

 7: **y′←***c*_*i*_**y**^**i**^ + *c*_0_**y**^**0**^

 8: **y**^**0**^**←y′**

Since it is necessary to read in the data and perform multiplication and addition in a finite field, the encoding operation is responsible for most of the algorithm’s calculations. Step 9 in Algorithm 1 is where the encoding is performed; only two data blocks need to be encoded during this process. The total overhead involved in coding is proportional to the number of data blocks; therefore, MINC requires less computation for the encoding process than RLNC does.

Next, we analyze the mathematical properties of MINC. Since the exact characterization of network coding in MP2P network is extremely difficult to be obtained, approximated analysis is applied onto such protocols based on several assumptions. In every discrete time-step, each node selects a limited communication partners randomly but uniformly among all nodes with only one message transmitted [12]. Thus the following assumptions are maintained: (1) The number of data blocks received by an intermediate node satisfies a Poisson distribution with parameter *λ*. (2) The non-zero elements of a coding vector received by node *v* are uniformly distributed.

At time *t*, node *v* has *λt* blocks in its buffer; their encoding vectors are *d*_*1*_, *d*_*2*_, ⋯, *d*_*λt*_. $\bar{d}=\frac{\sum_{i=1}^{\lambda t}d_{i}}{\lambda t}$ denotes the average degree of all of the blocks in node *v*. Under the given conditions, the probability that node *v* selects **y**′ from *λt* blocks and **y′ ▷ y**^**0**^ is $P=1-{\left(1-\frac{\sum d_{i}}{\lambda t\cdot n}\right)}^{n-\bar{d}}$.

**Theorem 1:** The expectation of the number of times that a node first gets eligible block is less than $\frac{1}{P}$.

**Proof:** Denoted the number of times that a node first gets the eligible by *X*. It is easy to see that *X* is maximized when the degree of the benchmark block is $\bar{d}$. Because *X* has a geometric distribution with parameter *P*, the expectation is $E_{1}(X)={\sum_{j=1}^{\infty }i\cdot P\cdot \left(1-P\right)}^{j-1}=\frac{1}{P}$.

**Theorem 2:** The expectation of the degree of a new encoded block is less than $nP+\left(1-P\right)\bar{d}$.

**Proof:** According to the process of encoding with MINC, the degree of a generated block is maximized when the degree of the benchmark block is $\bar{d}$. Therefore, the expectation of the maximum degree is $E_{2}=\bar{d}+E\prime$, where *E*′ is the expectation of an increased degree when $d_{0}=\bar{d}$. The increased degree has a binomial distribution with parameters $\left(n-\bar{d},P\right)$ when $d_{0}=\bar{d}$; therefore, $E\prime =kC_{n-\bar{d}}^{k}P^{k}{\left(1-P\right)}^{n-\bar{d}-k}=\left(n-\bar{d}\right)p$, $E_{2}=nP+\left(1-P\right)\bar{d}$.

## Performance Evaluation

In this section, performance evaluation of RLNC, sparse NC and MINC encoding algorithms is launched comparatively for different conditions. To be specific, the algorithms are evaluated for their encoding speed, data distribution speed in dynamic networks. To evaluate the performance of the MINC algorithm, a simulator is constructed that afford the observation and measurement of the algorithm performance under a variety of conditions. The basic simulation parameters are chosen to model a dynamic network consisting of a collection of mobile nodes moving in an area with a fixed size.

The nodes’ motion is modeled with the modified random waypoint model [13]. All simulations are carried out in a rectangular area of 1500m*500m, the results of which are averaged for at least 100 simulation runs. Besides, node speeds are randomly distributed between 2m/s and 15m/s. At the start of every simulation, each node is initially placed at a random position within the simulation area and randomly chooses a new location as its destination. After reaching the destination, the node waits for a given pause time and starts moving towards some other randomly chosen destination.

To directly compare the performances of the coding algorithms on the basis of experimental results, a communication channel is configured with a high rate and uniform connection delay, with the influences of other factors ignored on the underlying network in that the simulation is for encoding performance only, not for a thorough performance comparison. Other specific experimental parameters settings are shown in Table 1.

**Table 1:** The parameters used in the simulations.

parameter	value
file size	100,200MB
block size	64-2048KB
nodes	50
communication distance	100m
pause time	0-3s

### Comparison of the encoding speeds

**Fig 2:**
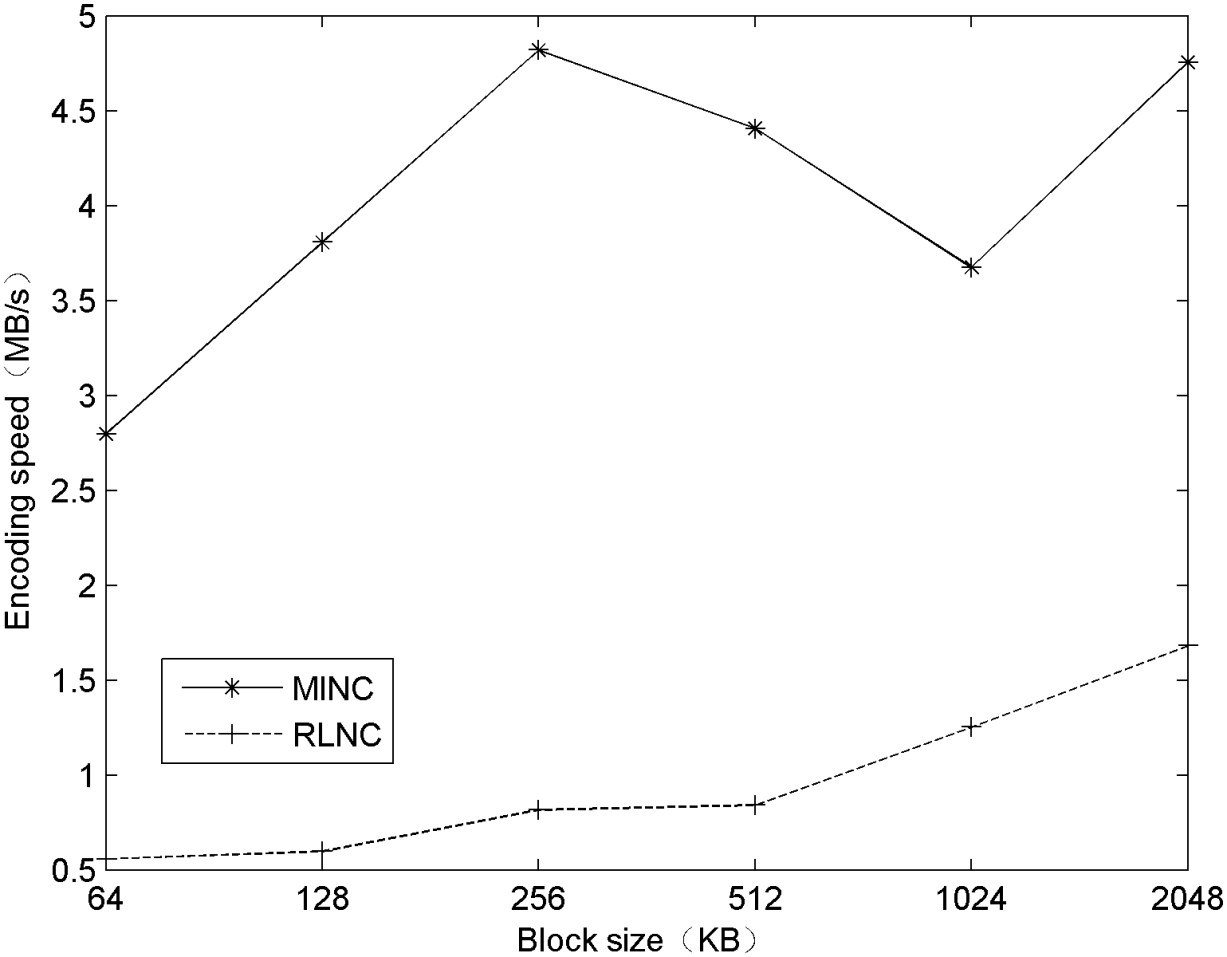
A comparison of the encoding speeds.

The basic idea runs as follows. The total time required is recorded to generate a certain number of encoded blocks of different sizes using the MINC and RLNC. The time required is recorded after the 200 MB encoded block has been generated using RLNC and MINC, and then, the average encoding speed is calculated. The results are shown in Fig 2.

From the results shown in Fig 2, it is clear that MINC is faster than RLNC. The maximum encoding speed of the MINC algorithm exceeds 4.5 MB/s, which satisfies the requirements of general dynamic network transmission. In the same hardware environment and with the same data block size, the encoding speed of the MINC algorithm is significantly greater than that of the RLNC algorithm. In addition, as the figure shows, the encoding speeds have the same trend with respect to the block size; as the block size increases, the number of blocks decreases, which reduces the amount of switching between selecting coefficients and encoding, therefore, increases the encoding speed. However, the curves have inflection points when the block size is 256 KB, which may be due to the system’s cache size, access control, or other factors.

### Comparison of the data distribution speeds

**Fig 3:**
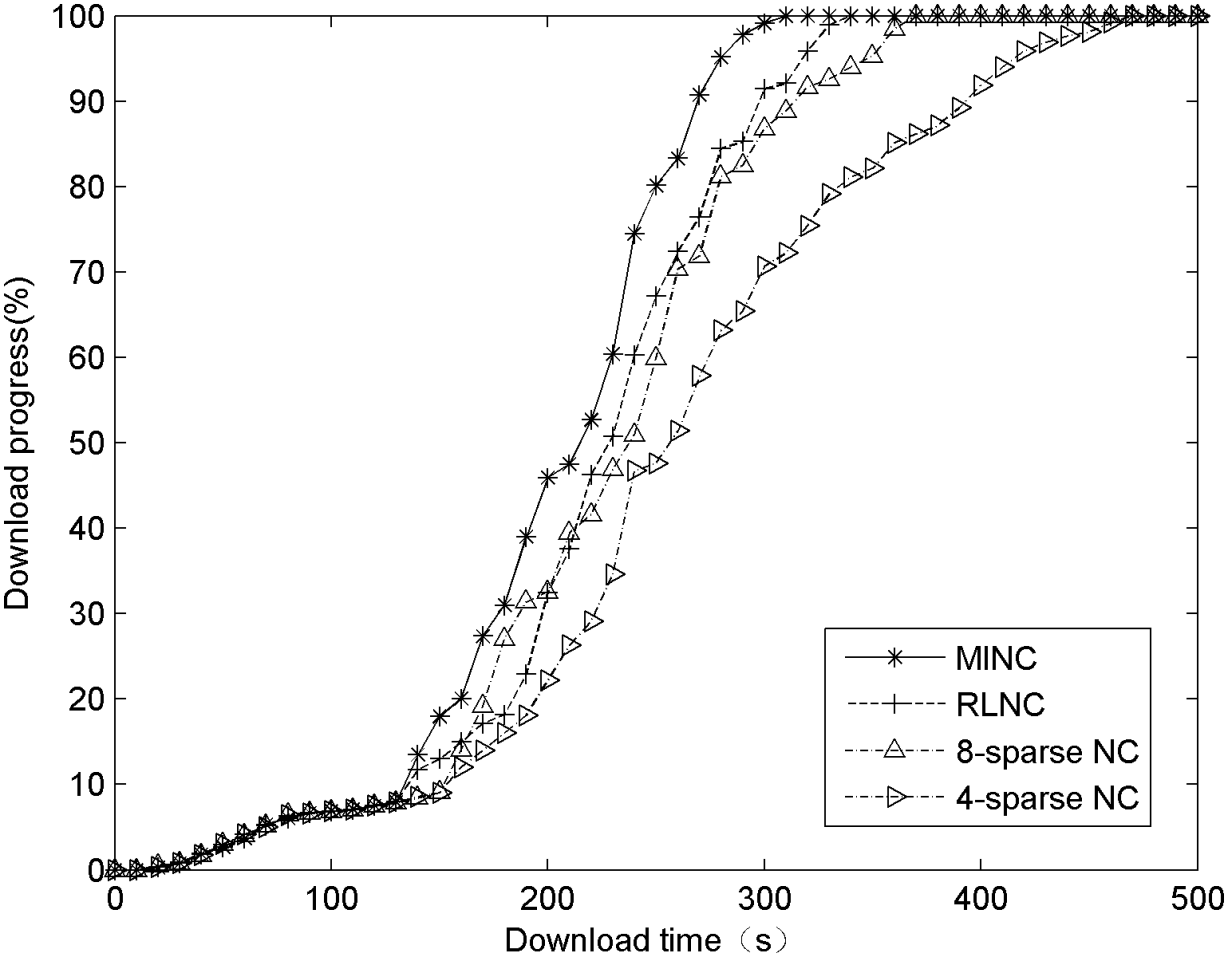
A comparison of the data distribution speeds.

The speed of data distribution is an important index of an algorithm’s performance. In addition to the encoding speed, the structure of an encoded block influences the velocity distribution, which is primarily shown in the linear correlations between blocks generated with different algorithms. A node encodes all of the data blocks in its domain of RLNC, which minimizes the probability of linear dependence; in contrast, a sparse encoding scheme increases the probability because it reduces the amount of data involved in the encoding process. In a comparative experiment, the file size is set to 100 MB and the block size to 256 KB and the results are compared between RLNC, 8- sparse NC, 4-sparse NC and MINC. The results are shown in Fig 3.

In Fig 3, the curves can be divided into three phases. In the first phase, zero or only a small number of data blocks are cached in nodes; therefore, data distribution begins slowly. In the second phase, which is the data distribution phase, most of contents are distributed. In the third phase, the target node receives a large amount of data, which increases the probability of linear dependence between new blocks and other blocks, yielding the decrease in distribution speed. In the second phase, the encoding speed is the primary factor influencing the distribution speed. The encoding speed of MINC supports the distribution speed more strongly than that of RLNC; 4-sparse and 8-sparse NC also perform well. The differences in the performances of the algorithms are very apparent in the third phase; the speed of the MINC algorithm decreases slightly, that of the RLNC algorithm is essentially stable, and that of fixed density encoding decreases significantly, that of especially 4-sparse NC. The experimental data reveal that the MINC algorithm distributes data approximately 7% faster than it encodes data completely.

**Fig 4:**
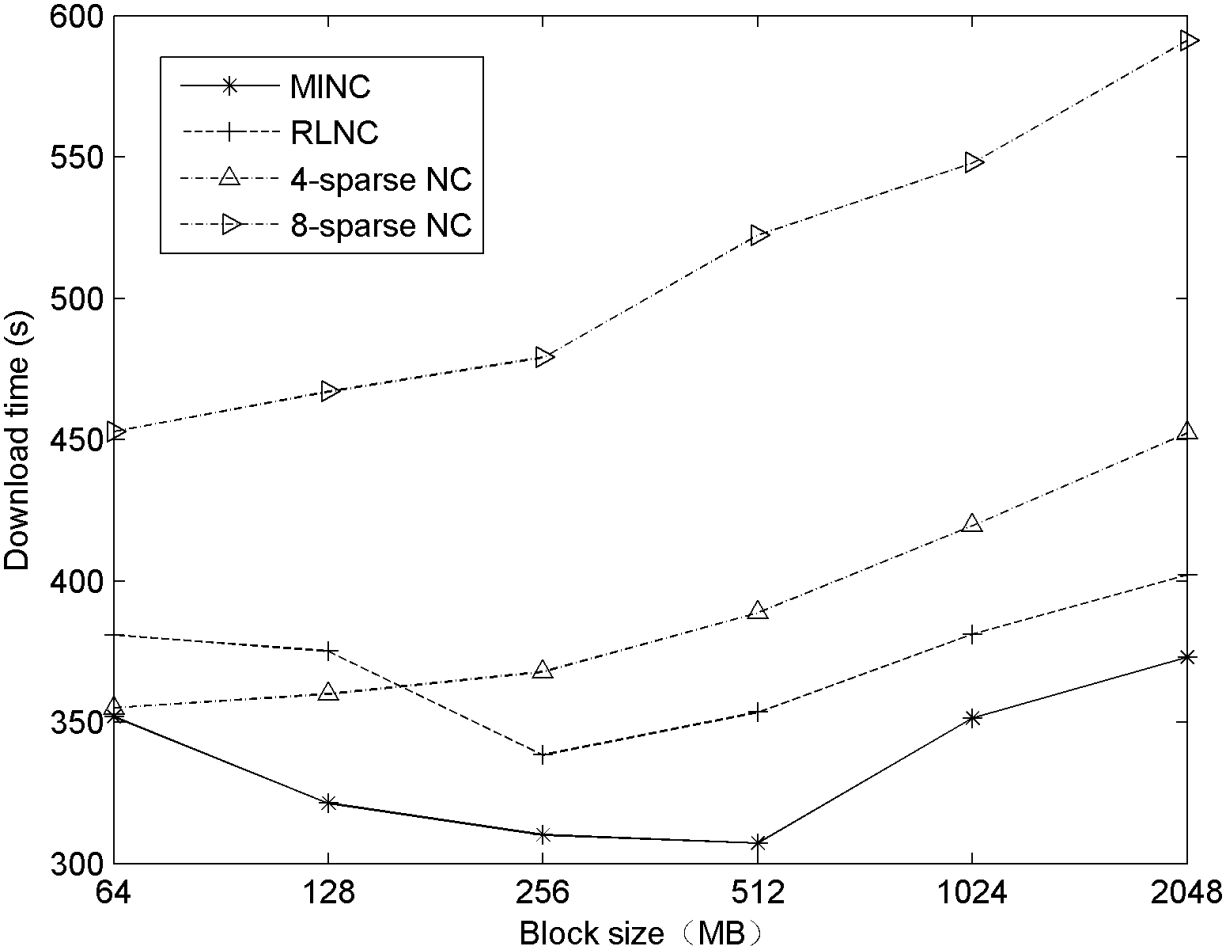
The effect of the block size on the distribution speed.

To study the effects of different block sizes on the distribution speed, a set of experiments is conducted on a 100 M file with a block size between 64 KB and 2048 KB. The results are shown in Fig 4.

From Fig 4, it is shown that the curves corresponding to MINC and RLNC are similar to the "V" glyph when the block size increases, which betrays that the speeds of the two algorithms affected the encoding speed and the structure of the encoding vector. The total distribution times of 4-sparse NC and 8-sparse NC tend to increase because the number of blocks involved in encoding is fixed; therefore, the speed is primarily affected by the construction of the encoding vector. When the total number of blocks is reduced, the probability of linear dependence between blocks increases, and so does the download time.

From these results above, it can be concluded that MINC performs better for various block sizes in the modified random waypoint model. Therefore, MINC is better suited to a dynamic network environment than other encoding algorithms.

## Conclusions

Although there are numerous encoding algorithms for network coding, many of them suffer from computational complexity or a lack of distribution efficiency. This paper aims to investigate the feasibility of reducing the computational complexity. To leverage the potential of network coding and keep the computational complexity low, a MINC scheme is proposed in which the encoder selects blocks to be encoded on the basis of the nonzero elements of the encoding vector. Since only two blocks are involved in the generation of new blocks, the computational complexity remains low. The effectiveness of the proposed coding scheme on the computational complexity in a dynamic network is demonstrated by means of simulations and performance comparisons. In the tradeoff between computation complexity and file sharing effectiveness, MINC ranks as an excellent compromise among the other schemes.

## Supporting Information

S1 Fig A comparison of the encoding speeds. (TXT)

S2 Fig A comparison of the data distribution speeds. (TXT)

S3 Fig The effect of the block size on the distribution speed. (TXT)
